# Reaching the Frail Elderly for the Diagnosis and Management of Atrial Fibrillation—REAFEL

**DOI:** 10.3390/ijerph20186783

**Published:** 2023-09-19

**Authors:** Carsten Bamberg, Caroline Thorup Ladegaard, Mathias Aalling, Dorthea Marie Jensen, Christoffer Læssøe Madsen, Sadaf Kamil, Henrik Gudbergsen, Thomas Saxild, Michaela Louise Schiøtz, Julie Grew, Luana Sandoval Castillo, Iben Tousgaard, Rie Laurine Rosenthal Johansen, Jakob Eyvind Bardram, Anne Frølich, Helena Domínguez

**Affiliations:** 1 Cardiology Department Y Bispebjerg and Frederiksberg Hospital, Capital Region, 2000 Frederiksberg, Denmark; carstenbamberg@gmail.com (C.B.); carolineladegaard@hotmail.com (C.T.L.); dortheamj@gmail.com (D.M.J.); christoffermadsen01@gmail.com (C.L.M.); sadafkamil88@gmail.com (S.K.); 2 Department of Biomedicine, University of Copenhagen, 1165 Copenhagen, Denmark; 3 VihTek Research Center for Welfare Technology Capital Region, 2600 Copenhagen, Denmark; mathias.aalling@regionh.dk; 4 Section of General Practice, Department of Public Health, University of Copenhagen, 1165 Copenhagen, Denmark; henrik.gudbergsen@sund.ku.dk; 5 Grøndalslægerne Godthåbsvej 239a, Vanløse, 2720 Copenhagen, Denmark; saxild@mac.dk; 6 Center for Clinical Research and Prevention Bispebjerg and Frederiksberg Hospital, 2400 Copenhagen, Denmark; michaela.louise.schioetz@regionh.dk (M.L.S.); julie.grew@regionh.dk (J.G.); 7 Geriatrics Department, Bispebjerg Hospital, 2400 Copenhagen, Denmark; luana.sandoval.castillo@regionh.dk; 8 Department of Quality and Education, Bispebjerg and Frederiksberg Hospital, 2400 Copenhagen, Denmark; iben.tousgaard.01@regionh.dk (I.T.); rie.laurine.rosenthal.johansen@regionh.dk (R.L.R.J.); 9 Department of Health Technology, Digital Health, Personalized Health Technology, Technical University of Denmark, 2800 Copenhagen, Denmark; jakba@dtu.dk; 10 Innovation and Research Centre for Multimorbidity, Slagelse Hospital, Region Zealand, 4180 Sorø, Denmark; anfro@sund.ku.dk; 11 Section of General Practice, Faculty of Health and Medical Sciences, University of Copenhagen, 1165 Copenhagen, Denmark

**Keywords:** sensor, Holter, monitoring, atrial fibrillation, health professionals, frail elderly, cross-sector collaboration, CardioShare model, C3+

## Abstract

BACKGROUND: Frail elderly patients are exposed to suffering strokes if they do not receive timely anticoagulation to prevent stroke associated to atrial fibrillation (AF). Evaluation in the cardiological ambulatory can be cumbersome as it often requires repeated visits. AIM: To develop and implement CardioShare, a shared-care model where primary care leads patient management, using a compact Holter monitor device with asynchronous remote support from cardiologists. METHODS: CardioShare was developed in a feasibility phase, tested in a pragmatic cluster randomization trial (primary care clinics as clusters), and its implementation potential was evaluated with an escalation test. Mixed methods were used to evaluate the impact of this complex intervention, comprising quantitative observations, semi-structured interviews, and workshops. RESULTS: Between February 2020 and December 2021, 314 patients (30% frail) were included, of whom 75% had AF diagnosed/not found within 13 days; 80% in both groups avoided referral to cardiologists. Patients felt safe and primary care clinicians satisfied. In an escalation test, 58 primary-care doctors evaluated 93 patients over three months, with remote support from four hospitals in the Capital Region of Denmark. CONCLUSIONS: CardioShare was successfully implemented for AF evaluation in primary care.

## 1. Introduction

The cumulative prevalence of AF in Denmark is 3.0% [1], increasing with age [2,3] and causing important morbidity [4]. Especially in the frail elderly, the risk of unplanned hospitalization and adverse outcomes is increased [5,6,7,8]. They have a particularly high risk of stroke, which can be prevented with timely anticoagulant medication [9,10,11]. Nonetheless, frail elderly patients with AF are less likely to be treated with anticoagulants and managed with rhythm control than other patients [12].

The detection of AF can be challenging, as approximately 30% have no symptoms [13,14,15]. The overall objective of this project was to develop and implement a collaboration model for general practitioners (GP) and hospital cardiologists that allows for evaluation of frail elderly patients to timely diagnose atrial fibrillation (AF) at less of a burden for the patient.

In the conventional diagnostic workup on patients suspected to have AF paroxysms, the GP refers the patients to the hospital cardiologists for evaluation, including heart rhythm monitoring. Patients are usually scheduled for several meetings at the outpatient clinic before the diagnosis is confirmed or rejected and treatment eventually initiated: (1) initial consultation with a cardiologist, (2) receiving a Holter and starting monitoring, (3) delivering back the Holter, (4) consultation with a cardiologist to become informed on the results and next steps in the diagnostic process and treatment. This is cumbersome, especially for patients who are physically or mentally frail, and who often need help for transportation. These patients tend not to be referred or terminate the diagnostic workup prematurely, still suffering the risk of subsequent stroke due to possible non-diagnosed AF. The REAching the Frail ELderly study (REAFEL) was designed to simplify the workup process for diagnosis and management of suspected AF, making it possible to conduct Holter monitoring in a primary care setting and thus aiming to avoid a need for referral to the outpatient clinic. In REAFEL, GPs could use a simple continuous cardiac rhythm-monitoring device (Holter) and received remote support from cardiologists to interpret the results and to guide decisions on the need for referral to further cardiologist evaluation and on choice of adequate anticoagulation therapy. In this assessment, the dialogue between the cardiologist and the GP can be crucial in making the correct decision, as the GP often knows the patient’s risk of bleeding and treatment preferences. Additionally, in REAFEL patients could have video consultations with the cardiologist in cooperation with their GP.

We hypothesized that GPs could initiate Holter monitoring and reach a conclusion adequately and safely, thus minimizing the need for referral to the cardiology outpatient clinic. We also wanted to explore whether patients and GPs would be confident and satisfied with this workup.

The Danish company Cortrium ApS (Høje-Taastrup, Denmark) is one of the partners that received the grant from the Danish Innovation Foundation (grant 6153-00009B) and provided C3+ sensors, which are compact three-channel (Holter) sensors that can be connected to conventional electrodes and are easy to manage without special skills.

## 2. Materials and Methods

The entire REAFEL project followed the frame of a complex intervention [16] that comprises a pilot study, a pragmatic cluster randomization study, and an escalation phase.

We developed a workup for the diagnosis and management of AF in a pilot study together with one GP clinic with six GPs who received support from the department of cardiology of Bispebjerg and Frederiksberg Hospital between 2019 and 2020, and called it the CardioShare model [17].

For reference to assess workup time from usual referral until report conclusion, we collected data on all referrals to Holter monitoring at the hospital’s outpatient department during a randomly chosen month (January 2019) and compared it with the workout time we used with the CardioShare model in the pilot study.

We explored how Holter monitoring would be used by GPs with and without remote support from a cardiologist in a pragmatic cluster randomization study, where all GPs received a compact three-channel Holter monitor (C3+, provided by Cortrium ApS, Høje-Taastrup, Denmark). GP clinics were then randomized as a cluster, including all doctors in the clinic, to receive remote support from a cardiologist (CardioShare arm) or to decide at their own discretion which patients to monitor and further refer to the cardiology outpatient clinic (non-CardioShare arm).

The trial is registered in www.clinicaltrials.gov (NCT04162548) and meets the criteria for a Pragmatic Explanatory Continuum Indicator Summary (PRECIS) as follows [18,19]:

(1) Patients evaluated in the project are the same as those evaluated in conventional practice. (2) Patient inclusion happened in connection with an ordinary consultation. (3) The healthcare staff treating the patients in the project (GPs, and cardiologists) were the same as those treating patients routinely. (4) The resources used in the project (GPs, cardiologists, and nurses at the outpatient department, who evaluated the Holter monitor recordings) were the same as those conducting evaluations in the conventional process at the outpatient department. (5) The proposed CardioShare model was intended to be as flexible as the conventional outpatient diagnostic evaluation. (6) Follow-up of patients was the same as for conventional evaluation, except that study patients were asked to participate in study-specific interviews. (7) The primary outcome was clinically relevant for the usual patient evaluation process. In this project, the primary outcome was the number of (frail) patients who completed workout from deciding to monitor the heart rhythm to when adequate treatment was initiated based on a conclusive diagnosis. (8) All the data collected in the project were analyzed as part of the primary outcome.

### 2.1. A Priori Calculation of the Population of Interest

We calculated whether we could manage to perform Holter monitoring in a feasible number of frail patients to prevent a stroke with timely initiation of anticoagulation therapy.

In 2015, stroke was the cause of 3.4% of all somatic hospital admissions for patients older than 85 years [20]. When AF was identified, there was a total recurrence of stroke of 14% in this group [21]. With a total number of admissions of 37.998 in 2014, we calculated that 1.808 recurrences of stroke were preventable with timely anticoagulation therapy. Since anticoagulation medicine can prevent 1/3 of strokes related to AF in the elderly [7], in 2014, there were 600 preventable cases of stroke in Denmark. We conducted a similar calculation for the Capital Region, where there are 2300 admissions/year for stroke, which was a stable number in three consecutive years until 2016. In total, 1300 of these patients were admitted to BHF, which corresponds to 350 patients suffering from preventable strokes. With a 20% diagnostic identification of AF, and an efficacy of anticoagulation of 3 patients out of 10 in the frail elderly group (CHADS-VASc score of 4 or more), we calculated that if 10 frail patients would undergo Holter monitoring weekly, we could monitor 350 patients within one year, which was a feasible process.

### 2.2. The CardioShare Model

From February 2020 to December 2021, nine GP clinics were gradually included as clusters and randomized 1:1 into a CardioShare/intervention group and non-CardioShare/control group. The inclusion of the clinics was based on outreach meetings and networking. Each clinic was randomized as a cluster, regardless of the number of GPs working in the clinic, and all patients were managed from the clinic according to the allocated randomization. All Holter reports were evaluated and approved by the hospital’s cardiologists, who could review recordings on demand.

**Figure 1:**
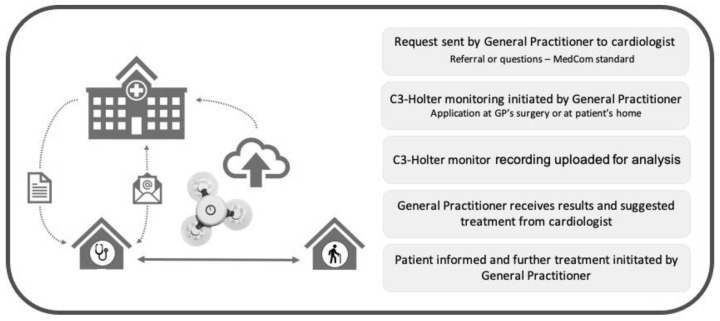
The CardioShare model.

To receive remote support by cardiologists, GPs in the intervention (CardioShare) group used the national platform for cross-sector communication MedCom (www.medcom.dk/standarder, accessed on 13 September 2023). The cardiologists confirmed the indication for monitoring or asked for further information before the GP initiated Holter monitoring. Length of monitoring varied from one to seven days, according to the cardiologist’s advice. Subsequently, all recordings were uploaded to a cloud-based analysis platform provided by Cortrium. The cardiologist received a notification from Cortrium when recordings were analyzed and a report was available, then forwarded the report to the GP along with advice on how to manage the patient according to the findings. The GP informed the patients if further evaluation or treatment at the cardiology department was recommended. With patient consent, the GP would then send a message to the cardiologist, who could schedule an appointment with the patient without additional referral. The GP could ask the cardiologist to communicate directly with the patient by means of video or phone consultation. Figure 1 provides an overview of the process.

The procedure for monitoring was the same for GPs in the control (non-CardioShare) group, but they initiated monitoring as they deemed needed, and solely decided patient management, including whether to refer the patient to a cardiologist for further evaluation. In this group, the monitoring reports from the cloud-based analysis platform were sent to the GPs without advice on patient management, while GPs were guided remotely by a cardiologist, i.e., using the CardioShare model (intervention group).

Observation parameters recorded were: (1) CardioShare: number of days from the GP’s first message to a cardiologist to results and recommendations sent to the GP by a cardiologist, (2) non-CardioShare: number of days from initiation of recording to results and recommendations sent to the GP by a cardiologist. To standardize our statistics, all delayed cases as well as those with more than one Holter recording were not considered. Our results were compared to the cohort of patients (*n* = 117) who attended the outpatient department at Bispebjerg-Frederiksberg Hospital throughout January 2019 (pilot project phase) for initiation of Holter monitoring as a reference for usual care, measuring the number of days from date of referral to results and recommendations given to the patient and/or sent to the patient’s GP (whichever was earliest).

**Inclusion criteria:** Patients with suspected AF and patients with known AF where the GP needed information for heart rate control or to assess AF-burden. We encouraged GPs to include patients they considered frail elderly, but inclusion was liberal in order to explore other patient groups being considered relevant from a primary care perspective. To define “frailty” we used a modified version of the simple criteria described for the age group at the greatest risk of stroke from suspected AF (Table 1) [22,23,24]. We classified indications for Holter monitoring according to Table 2.

**Exclusion criteria:** Patients younger than 18 years and patients who did not provide written informed consent were excluded from the project. Likewise, patients with suspicion of severe arrhythmia who needed to be referred to a cardiologist and who could attend the outpatient department were excluded.

### 2.3. Patient Reported Experiences

To explore participating patients’ experiences of the diagnostic process, all patients who completed a Holter monitor recording were contacted by phone and asked to answer a short survey. When patients did not complete the survey, we recorded the reason as: no contact data available in the patient’s electronic journal, the patient did not answer the phone after at least three attempts, the patient did not want to participate, or the patient did not understand the survey’s questions due language issues or impaired cognitive function.

**Figure A1:**
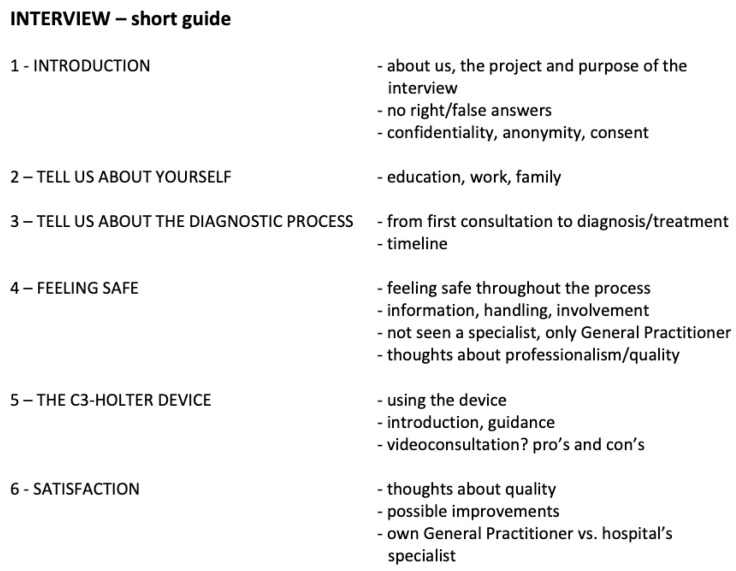
Short version of interview guide.

To further explore the data collected through the survey, we randomly selected 20% of the patients among those who completed it and invited them to participate in a semi-structured interview concerning their experiences during the diagnostic process. To minimize a biased selection of participants for these interviews, we compiled a list of all cases sorted by inclusion date, and invited every fifth patient in the list for an interview. If the invitation was not successful, the next patient on the list was invited. The semi-structured interviews were conducted according to a guide that included a list of the topics that we wanted to explore during the interview (Figure A1). Throughout every conversation, the interviewers encouraged the respondents to speak freely and only asked questions to elaborate within the topics of interest. The conversations were recorded and evaluated continuously until sufficient data were collected, i.e., data saturation, when interviews of two consecutive patients did not provide any new aspects within the fields of topics. Interview recordings were subsequently transcribed verbatim. Two researchers independently coded all interviews before analyzing the patients’ narrations and organized the large amount of text in a concise summary of key results, as described by Erlingsson and Brysiewicz [25].

### 2.4. Participating Health Care Professionals’ Experiences

In addition to patient interviews, we also invited GPs and nurses to participate in interviews to explore their experiences with the CardioShare model. We conducted 14 interviews with a total of eight GPs and six nurses from both clusters. All conversations were recorded and analyzed using the same method as described for patient interviews.

## 3. Results

### 3.1. Cluster Randomization Study

#### 3.1.1. CardioShare Versus Non-CardioShare

**Figure 2:**
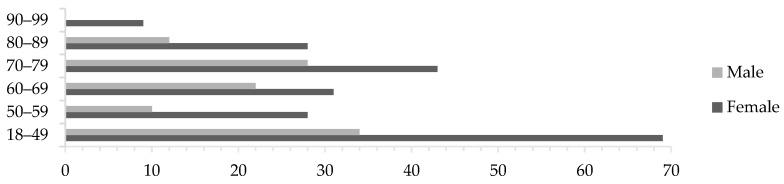
Distribution of age among all participants by gender; *n* = 314.

From February 2020 to December 2021 a total of 314 patient cases were evaluated; 122 were randomized CardioShare and 111 to non-CardioShare. The remaining 81 cases were enrolled during the same period but by GPs at our feasibility phase clinic, resulting in a non-randomized mixture of CardioShare and non-CardioShare patients. Predominantly female patients participated in the study, across all ages (Figure 2), with a median age of all participants of 62.5 years. In total, 85 patients (27%) were classified “frail”, 61 being female and 24 being male. In twelve cases, Holter recording was repeated and one patient underwent a third recording. User failures occurred in eight cases, mostly due to a lack of routine among the staff in the start-up phase of the project. In four cases, the cardiologists recommended a follow-up recording based on the patient’s symptoms. The patient who underwent a third recording did so due to new symptoms six months after the initial recordings.

There were recorded delays in the workout process in 64 cases; 6 caused by the patient, 33 caused by the GP, and 25 by the cardiologist.

**Figure 3:**
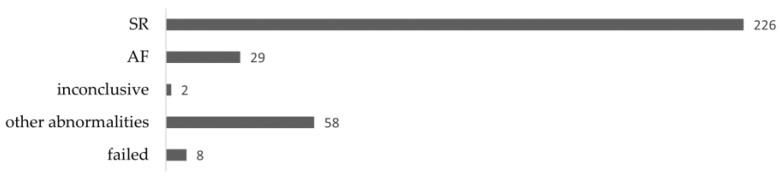
Results of Holter recordings; *n* = 323.

Among a total of 323 recordings during the cluster-randomized main study, 29 patients (9.0%) were diagnosed with AF. The majority, 226 recordings (70.0%), showed normal sinus rhythm, whilst 58 (18.0%) showed other abnormalities, two were inconclusive, and eight recordings failed. In 257 cases (79.6%), no further involvement of the cardiologist was needed, and the GP took over further treatment if necessary/as advised. In total, 46 recordings (14.2%) resulted in the patient’s referral to a cardiologist for further diagnostics. In 16 cases (5.0%) cardiologists advised to perform another Holter recording, 8 of which were due to failed recording (Figure 3).

Of the 58 recordings that showed other abnormalities than AF, 33 (56.9%) resulted in referral to a cardiologist for further diagnostics, whilst 21 cases (36.2%) could be handled by the GP without further involvement of other specialists. In three cases, the cardiologists advised to conduct another recording, and in one case, the protocol was violated (non-CardioShare cluster) as the patient’s GP was advised to change medication.

**Figure 4:**
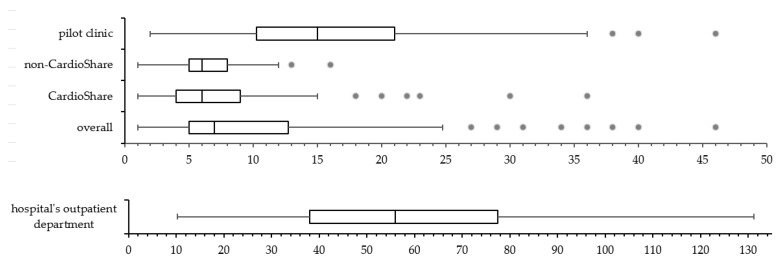
Duration of diagnostic process from decision to results (days). Top: cluster randomization study (pilot clinic that continued to include patients during same period of time; non-CardioShare clinics; and CardioShare clinics; overall *n* = 314 patients). Bottom: comparison cohort of patients managed as usual in the hospital’s outpatient department; *n* = 117 patients. Please be aware of the scale difference between top and bottom parts of the figure.

During the pilot phase (sample data collected throughout January 2019), a total of 117 patients were referred to the hospital’s outpatient department to undergo Holter recording. The duration of the diagnostic process was measured from the date of referral to the date when the cardiologist gave the results to either the patient’s GP or the patient themselves. The mean duration of the diagnostic process for AF was 63 days, ranging from 10 to 224 days, and 75% of all patients were diagnosed within 78 days (Figure 4, bottom). In comparison, in the REAFEL study, the mean duration of the workout for diagnosing AF was shorter (from 1 to 25 days) for all patients, as 75% of all patients had AF diagnosed/not found within 13 days. Patients who during the main study were included by our pilot phase clinic are shown separately (Figure 4, top).

#### 3.1.2. Escalation Test

After the cluster-randomized study ended (December 2021) a workshop was held where more GPs and cardiologists from other hospitals in the Capital Region of Denmark were invited to discuss the experiences and how CardioShare could be implemented for diagnosis and management of AF. There was a consensus that GPs could initiate Holter monitoring and interpret the results provided if the cardiologist continued to respond within a short time to the requests from GPs with doubts about the indication for Holter monitoring, or about the results of the report or the following evaluation and/or treatment of the patients. Four cardiology sites with hospital cardiologists and two private practicing cardiologists used the CardioShare model to support 58 GPs distributed in 13 clinics in the escalation test from February to April 2022. In total, 93 patients were evaluated by their GPs using C3+ sensors and supported through the CardioShare model. The Cardiology Council approved this procedure as an additional standard of care method in the Capital Region of Denmark.

### 3.2. Survey

**Figure 5:**
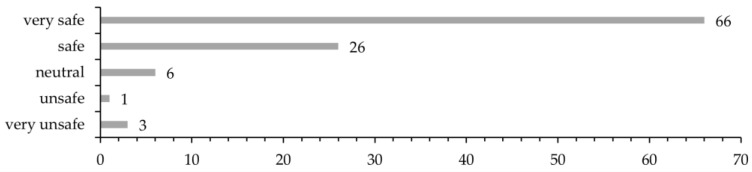
Survey: How safe did you feel during the whole diagnostic process? *n* = 102; very unsafe = 3 (2.9%); unsafe = 1 (1.0%); neutral = 6 (5.9%); safe = 26 (25.5%); and very safe = 66 (64.7%).

**Figure 6:**
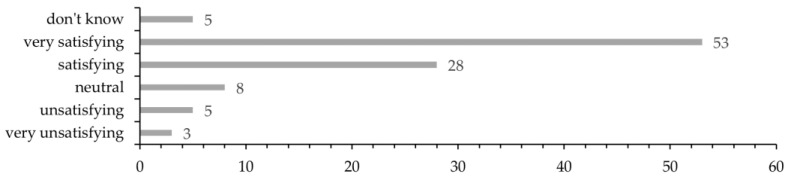
Survey: Evaluate the organization of the diagnostic process (*n* = 102); don’t know = 5 (4.9%); very satisfying = 53 (52.0%); satisfying = 28 (27.5%); neutral = 8 (7.8%); unsatisfying = 5 (4.9%); and very unsatisfying = 3 (2.9%).

In total, 160 patients were asked to participate in the survey, of whom 102 (63.8%) accepted. The patients were asked how safe they felt from the time they saw their GP due to their symptoms until being explained the outcome of the Holter recording. In total, 90.2% of the patients felt either safe (25.5%) or very safe (64.7%), while 3.9% of the patients felt unsafe or very unsafe (Figure 5). A total of 81 patients (79.4%) were either satisfied (27.5%) or very satisfied (52.0%) with the organization of the diagnostic process. A few did not answer this question (4.9%), and 7.8% of the patients were unsatisfied (4.9%) or very unsatisfied (2.9%) (Figure 6). We were aware that patients tend to answer less critical when responding the survey’s closed questions regarding overall satisfaction with the workout process. Therefore, we addressed this specifically as an open question during the interviews.

The C3+ sensor was evaluated as user-friendly by 84.3% of the patients, whilst 4.9% had bad or very bad experiences with it. There is no association for this point with their level of satisfaction with the overall process. Hence, we can assume that the reasons for patients not being satisfied with the diagnostic evaluation process may be related to the doctor–patient relationship or to organizational matters.

When asked how worried the patients were about the symptoms for which they requested help from their GP, in total, 38 patients (37.3%) were either worried or very worried at that time. This number was halved at the end of the diagnostic process (15.7% were worried or very worried), while 25 patients (24.5%) stated the same level of concern before and after diagnostic evaluation. In depth analysis showed that 8 of these 25 patients answered “not worried at all” in both cases, whilst other five patients (4.9%) answered “worried”, and three (2.9%) answered “very worried” to both questions. Five patients (4.9%) indicated a higher level of concern after the diagnostic process was fulfilled.

### 3.3. Semi-Structured Interviews with Patients

Patients’ narratives were analyzed as shown in Table A1, and after eleven interviews, we could identify three main themes (i.e., hypotheses to be confirmed or disproved): (1) patients experience a high level of professionalism and quality despite not being seen by a cardiologist; (2) the C3-Holter device is user friendly and easy to handle; and (3) being diagnosed by their own GP makes patients feel safe and secure. The main results within these three main themes are as follows.

#### 3.3.1. Main Theme 1: Patients Experience a High Level of Professionalism and Quality despite Not Being Seen by a Cardiologist

For most of the patients, it does not make any difference if they are only seen by their own GP and not a cardiology specialist. They are confident knowing that their GP and hospital’s cardiologists work together:

“I assume there is no big difference between the approaches. It’s just the consultation that’s different. I wasn’t nervous at all about quality”.

“I was told that cardiologists saw my recording. So, it actually was the specialists who did analyze it”.

“I think it is just fine that cardiologists give advice [on further treatment] to my GP”.

One of the patients would prefer to be seen by a cardiologist right away. Some patients mentioned that it is important for them to be referred by their GP to a specialist right away if their symptoms could be caused by a severe condition.

“It depends on the severeness. If I’m having heart pain or feeling that my heart skips beats, I’d prefer to be referred to cardiologists right away. I’m sure my doctor would do so”.

#### 3.3.2. Main Theme 2: The C3-Holter Device Is User Friendly and Easy to Handle

The device being very small, most patients forgot about it while wearing it. They had no problem wearing their clothes, and they did not change their everyday activities. The vast majority of them did not take the device off at all. Those who did, e.g., to take a shower, had no problems reattaching it.

“I did the usual things—went for a walk every day, played some golf”.

“It was only three days, so I let it be and just washed around it”.

“They explained how to replace the patches, and I am comfortable to do so”.

“After a shower, I took it back on without any problem”.

The most common problem reported by the patients was discomfort caused by itchy skin reactions to the standard electrodes.

“I didn’t pay any attention to the device before my skin became itchy”.

“When you’ve been wearing it for three days, your skin has become very itchy, and you look forward to get rid of the device”.

Some patients appreciated the possibility to set a marker on the recording whenever they experienced any symptoms.

“I quite liked the possibility to push the button when I was experiencing symptoms”.

“When I felt any kind of symptoms, there was this button I just could push to set a marker on the recording”.

#### 3.3.3. Main Theme 3: Being Diagnosed by Their GP Makes the Patients Feel Safe and Secure

Most of the patients feel safe and secure with their GP, but it depends on their interpersonal relationship, as mutual trust is built up over time.

“It depends on, which one of the GPs I’m seeing”.

“In the old days I felt safe, because you always saw the same doctor. They kind of knew your journal. Today there are many different [doctors/GPs]”.

“I think it’s just fine, if it works. I think it’s nice, because it does mean a lot to feel comfortable with the person [doctor / health professional] you’re seeing”.

One factor that makes the patients feel safe and secure is the continuity they experience with their own GP.

“My doctor knows me and my medical history. A disease course can be broad, and specialists only look at things from their own discipline’s perspective”.

Some patients perceive it to be time saving and easier than attending the outpatient department at the hospital.

“There’s always a lot of waiting time. I prefer to see my GP”.

“I think it was nice, that the recording could be started right away”.

“When I need to go to the hospital I do need transportation help. I must be ready 90 min before, and then there’s waiting time and everything. I think it’s quite difficult”.

Except the few patients that reported technical or procedural problems during the diagnostic process, the patients in general were very comfortable with only seeing their GP and not being referred to the outpatient department at the hospital, but it is important to inform the patents also if there are technical problems.

“I hadn’t got any feedback from my GP. That was strange”.

“I called them after two weeks, because I hadn’t heard anything yet. I was told that the recording was empty”.

### 3.4. Semi-Structured Interviews with GP’s and Nurses

Through analysis of the health care professionals’ narratives, three main themes (i.e., hypotheses to be confirmed or disproved) could be identified: (1) the C3+ sensor is user friendly and easy to use in a GP’s setting; (2) cooperation between GP and cardiologist is helpful and appreciated; and (3) patients benefit from Holter monitoring at their GP’s. In this section, we will present the main results within these themes.

#### 3.4.1. The C3+ Sensor Is User Friendly and Easy to Use in a GP’s Setting

Overall, the GPs were positive about using Holter monitoring as a diagnostic tool.

“I think it was quite straight forward. I just had to get used to how to send the correspondence [to the cardiologist] and what to write, but it was actually quite quick to get it sorted out”.

“There are so many sentences, with all sorts of things, and there are actually only four things you have to do, so you could actually […] make a much simpler guide”.

#### 3.4.2. Cooperation between GP and Cardiologist Is Helpful and Appreciated

GPs who used CardioShare had to become used to communicating with the cardiologists instead of issuing a referral, but they were very satisfied with the outcome of this communication.

“It has been, in other words, completely impeccable in every way”.

“When you have to interpret the results, it’s extremely nice to have a cardiologist backing you up”.

Furthermore, the GPs valued the possibility of conducting Holter monitoring at their clinic without referral to a cardiologist.

“Being able to do a Holter monitoring is like a function we have wanted, it has been difficult to access, […], and suddenly it has moved very close”.

“I think it belongs excellently here, at this level where you have professional cooperation along the way”.

#### 3.4.3. Patients Benefit from Holter Monitoring at Their GP’s

GPs were satisfied being able to perform Holter monitoring on frail patients who otherwise would not be able to be monitored, but also on patients other than frail.

“We have a huge population, we have two nursing homes for which we are primary doctors”.

“We’ve actually used it primarily for the unconclusive [patient group], where we thought they’re not quite candidates for a hospital cardiology evaluation process”.

“Health can be measured in many ways; whether they don’t get blood clots, or whether they are reassured in their fear of illness”.

## 4. Discussion

The main result of this study is that the CardioShare model was applicable for diagnosis and management of suspected AF in primary care clinics. The process was shorter than in usual care, it required fewer referrals to the cardiology outpatient clinic, thus being less of a burden for the patients and reducing the capacity needed in the hospital’s outpatient department. Another study also showed that teleconsultation may increase access to cardiology evaluation in underserved populations, while reducing in-person referrals [26].

The aim of the study was to facilitate the management of frail elderly under the suspicion of AF, and it is remarkable that two of the first 20 patients included by one of the GPs needed a pacemaker. These patients were considered frail and refused to be referred to the cardiology outpatient department for Holter monitoring, but accepted it to be performed by their GP, and to have a pacemaker implanted. Nevertheless, only 30% of all included patients fulfilled our frailty criteria (Table 1). This is a weakness inherent in pragmatic implementation research. At the same time, the liberal inclusion that was accepted in the study reflects the true need from GPs to evaluate other patients than those who are frail elderly. In 80% of all cases, GPs were able to complete evaluation for arrhythmia suspicion, which shows a great potential in avoiding referral of low-risk patients to the outpatient department, thus requiring fewer resources from the cardiology specialists. Nevertheless, no formal resource analysis was performed, which is a major limitation of this study.

Likewise, although patients reported a high level of satisfaction in the survey and following in-depth interviews, the study does not include standard quality-of-life questionnaires, which poses a limitation for comparisons with similar studies.

Although most patients were very satisfied with the diagnostic process being led by their GP, it cannot be concluded that the patients would be less satisfied by being referred to the hospital’s outpatient department since this study did not include any analysis of patient-reported experiences in a usual care group. Despite this limitation, it is remarkable that patients described the relationship with their GP as being crucial regarding whether they would prefer being referred to the hospital’s outpatient department. When implementing the CardioShare model, it is important to ensure that the patients receive feedback on the results. Some patients did not receive any feedback and were disappointed about it.

In general, the patients found the Holter device easy to use, e.g., not having any problems reattaching it to their chest after they took it off. Some patients experienced itching and chest discomfort while wearing it. Since the device uses the same regular electrodes as those used in standard Holter devices, the general advice is to choose the best-tolerated electrodes. A folder with FAQ and a troubleshooting guide for patients could be useful, and adequate training of all involved health care professionals is crucial, as most problems with utilization of the Holter device seemed to be start-up difficulties that could be avoided.

From the GPs’ perspective, Holter monitoring with an easy-to-use compact device and support from hospital-based cardiologists was appreciated. They were very satisfied with the CardioShare cross-sectoral cooperation model, as they felt that their patients experienced an easy and safe approach throughout the workup process. They reported a great need for this kind of collaboration as they find it cumbersome to gain access to Holter monitoring at the hospital’s outpatient department. This is similar to findings in observational studies that showed that teleconsultation support enhances primary care physicians’ confidence, capacity, and satisfaction rates [27,28].

## 5. Conclusions

The CardioShare model is feasible with Holter monitoring performed at the GP’s office or at the patient’s home. It makes it possible to reach frail patients suspected for AF, and the fact that 70% of all included patients were non-frail shows the true need from GPs to evaluate a greater variety of patients for AF. The model can be implemented for diagnosis and management of suspected AF in primary care and has the potential to reduce the number of referrals to the cardiologists and thus save resources.

## Figures and Tables

**Table 1:** Frailty criteria.

Frail elderly are aged ≥ 65 years and match at least one of the following:	
(1)	Need help with transportation to the hospital’s outpatient department.
(2)	Need help with personal hygiene.
(3)	Have reduced ability to walk (estimated to take > 5 s to walk 5 m).
(4)	Have unintentionally lost weight within the past year.
(5)	Have cognitive difficulties (dementia, memory deficits, aphasia, etc.).
(6)	Have social problems due to abuse, ethnic background, language, etc.

**Table 2:** Criteria for diagnostic evaluation.

(1)	Unresolved palpitations.
(2)	Unclear episodes of dizziness.
(3)	Doubt on heart rate control in new atrial fibrillation diagnosis.
(4)	Suspicion of other arrythmias in patients with known atrial fibrillation.

## Data Availability

Outcomes from the cluster-randomization study can be provided by the corresponding author upon request.

## Appendix B

**Table A1:** Analysis of interviews (example): transforming meaning units (quotes) into condensed meaning units without loss of core meaning; labeling the condensed meaning units with codes; grouping into categories; and expressing categories’ meaning as themes ^1^.

Meaning Unit	Condensed Meaning Unit	Code	Category	Theme ^1^
“It wasn’t heavy or impractical in any way to wear the device. It was quite easy and didn’t bother me at all. I worked, went for a walk, and many other things”.	Device is not bothering; easy to wear; activities as usual	Everyday life with C3 monitor	Comfort	The device is user friendly/easy to use.
“My skin became very itchy when wearing it [the device]”.	Patches causing itchy skin reaction	Source of annoyance		
“You are kind of nervous whether the device might fall off. It actually did when I was playing golf. It was hot, and I was sweating a lot”.	Device fell of due to sweating while playing golf.	Technical problems	Reliability	

^1^ Themes are hypotheses that can be confirmed or disproved.
